# Treatment of recurrent tracheal adenoid cystic carcinoma with a covered airway stent loaded with Iodine-125 seeds: a 5-year follow-up case report

**DOI:** 10.3389/fonc.2024.1450508

**Published:** 2024-11-19

**Authors:** Zhen Yang, Yafei Wang, Zhenhua Li, Shuang Geng, Yi Hu, Hongling Hu

**Affiliations:** Department of Respiratory and Critical Care Medicine, The Central Hospital of Wuhan, Tongji Medical College, Huazhong University of Science and Technology, Wuhan, Hubei, China

**Keywords:** tracheal adenoid cystic carcinoma, iodine-125, airway stent, bronchoscopy, case report

## Abstract

**Background:**

The treatment of recurrent tracheal adenoid cystic carcinoma (TACC), a rare pulmonary malignant tumor, typically involves bronchoscopic interventional therapy for patients ineligible for surgery or external radiotherapy. This report describes an innovative treatment approach for TACC, initially managed with interventional bronchoscopy and subsequently with a Y-shaped airway stent loaded with ^125^I seeds, following recurrence after 2 years.

**Case presentation:**

A 50-year-old man presented with intermittent coughing for 2 months and was admitted to the hospital after the discovery of TACC a month earlier. Preoperative bronchial artery embolization was performed to reduce the risk of bleeding, followed by bronchoscopic tumor resection in January 2017 using an electric loop at the base, complemented by argon plasma coagulation (APC) and cryotherapy. Although short-term outcomes were favorable, the long-term prognosis remained suboptimal. Due to tumor recurrence, airway tumor resection and implantation of the ^125^I seed stent were performed under bronchoscope guidance in early 2019. The stent was removed 5 months later. The patient tolerated the procedure well and exhibited no signs of relapse or progression during the 5-year postoperative follow-up period. This case illustrates the successful application of a stent loaded with ^125^I seeds for treating unresectable TACC.

**Conclusions:**

Treatment of TACC with a covered airway stent loaded with ^125^I seeds may be a safe, effective, and scalable method.

## 1 Introduction

Tracheal adenoid cystic carcinoma (TACC) is a rare pulmonary malignant tumor characterized by low-grade malignancy and slow growth. However, it is prone to local recurrence and distant metastasis. Primary tracheal malignant tumors represent approximately 0.2% of all respiratory tumors, with TACC constituting 10%–20% of these cases (1). TACC originates from glands within the tracheal and bronchial walls. Approximately two-thirds of TACC cases occur in the lower part of the trachea and at the origin of the left and right main bronchi, whereas the remaining two-thirds arise at the origin of the large bronchus. Despite its low-grade classification, TACC often leads to varying degrees of airway stenosis, resulting in dyspnea and potentially life-threatening complications. The current preferred treatment for TACC is surgical resection combined with radiotherapy; however, many patients are unable to undergo surgery or experience relapse due to extensive lesions, airway stenosis, late diagnosis, and poor tolerance.

Interventional bronchoscopy is currently used for patients who are unable to undergo surgery or external radiotherapy. Main methods include argon-ion coagulation, high-frequency electrocautery, cryotherapy, microwave therapy, laser treatments, stent implantation, balloon dilatation, intracavitary radiotherapy, and chemotherapy. However, this approach has a long treatment cycle, numerous complications, a high recurrence rate, and a poor long-term prognosis. This report describes an innovative treatment method for TACC, initially managed with interventional bronchoscopy and subsequently treated with a ^125^I seed airway-covered Y-shaped stent following recurrence after 2 years.

In theory, this innovative radioactive stent has the advantages of rapidly improving narrow airways and enabling continuous close-range radiotherapy to control malignant tumors. Therefore, a covered airway stent loaded with ^125^I seeds may be an option for treating TACC.

## 2 Manuscript formatting

### 2.1 Case presentation

**Figure 1:**
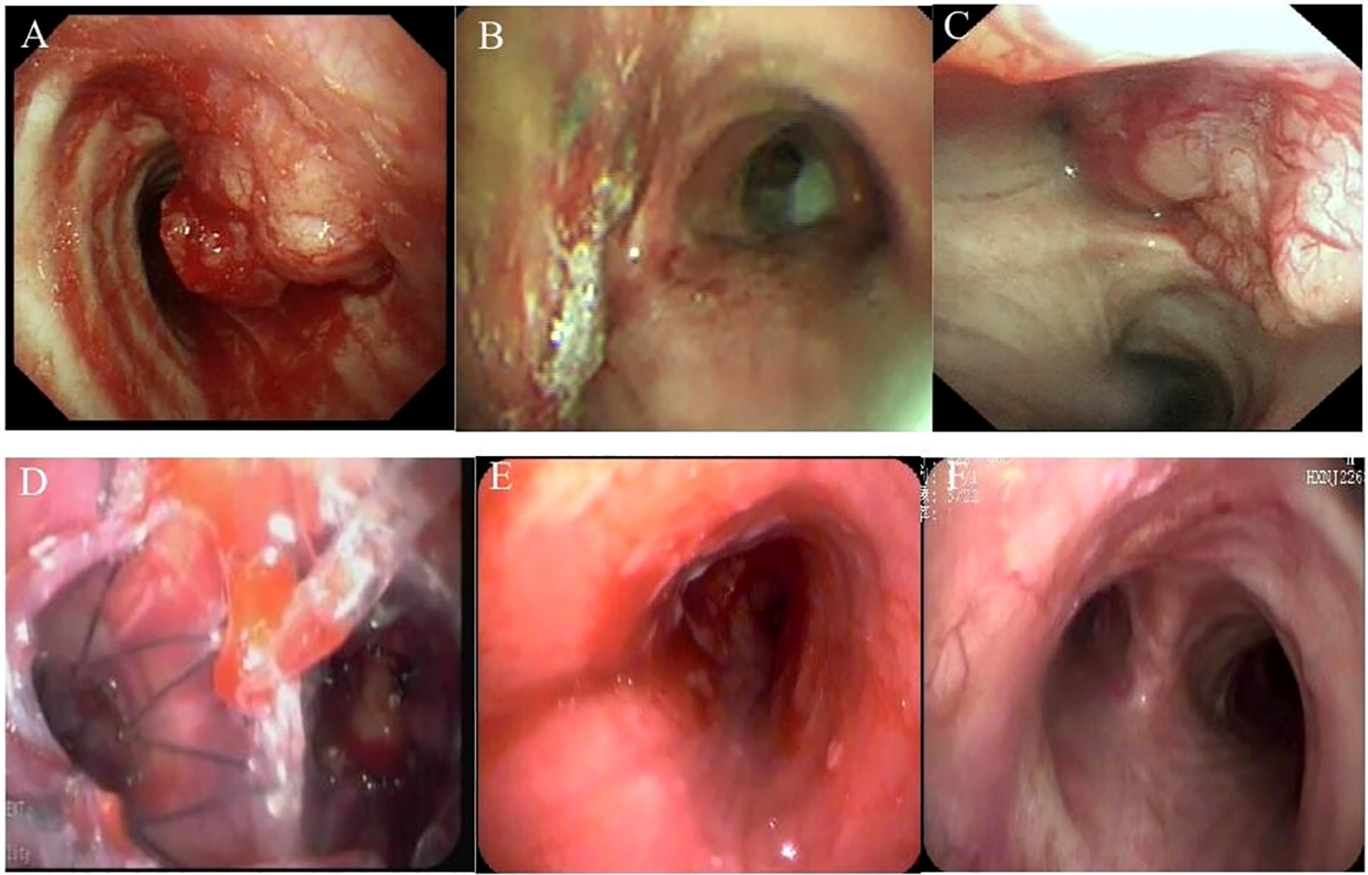
Bronchoscopy pictures. **(A)** 4 January 2017, tracheal adenoid cystic carcinoma (TACC) on the left side of the lower trachea near the carina. **(B)** 13 January 2017, TACC after interventional treatment by bronchoscopy. **(C)** 5 January 2019, TACC recurred. **(D)** 17 January 2019, ^125^I seed stent implanting. **(E)** 19 June 2019, after the ^125^I seed stent removing. **(F)** 17 August 2023, the main bronchus more than 4 years after removing the stent.

A 50-year-old man presented with intermittent coughing for 2 months and was admitted to the hospital after the discovery of new airway lesions 1 month earlier. A computed tomography (CT) scan at another hospital suggested a new organism in the bifurcation of the lower trachea. Bronchoscopy and subsequent biopsy revealed a TACC. The patient was referred to our hospital for further treatment on 4 January 2017. Physical examination revealed wheezing, slightly thickened breath sounds in both lungs, no obvious dry or wet rales, and an SPO2 at 95%. Urgent bronchoscopy revealed an obvious new biological entity on the left side of the lower trachea near the carina blocking the airway (**Figure 1A**).

**Figure 2:**
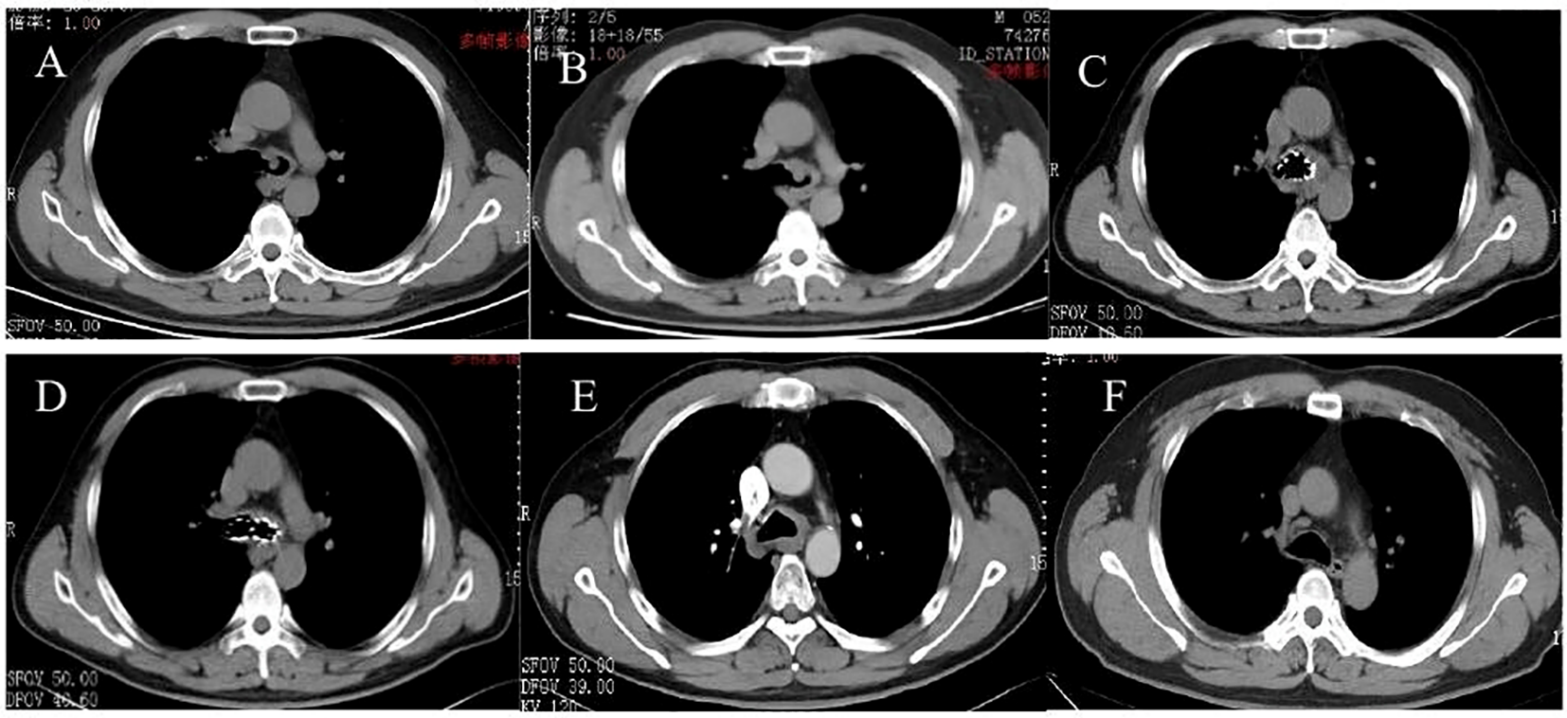
CT pictures. **(A)** 5 January 2017, tracheal adenoid cystic carcinoma (TACC) on the left side of the lower trachea near the carina. **(B)** 5 January 2019, TACC recurred. **(C, D)** 18 April 2019, ^125^I seed stent implanting. **(E)** 21 June 2019, after the ^125^I seed stent removing. **(F)** 17 August 2023, the main bronchus more than 4 years after removing the stent.

The findings were confirmed by chest CT (**Figure 2A**). To reduce the risk of surgical bleeding, bronchial arteriography and embolization were performed before bronchial interventional surgery due to the abundant blood supply to the neoplasm. The patient underwent intratracheal tumor resection under general anesthesia. Three pieces of tissue were removed with a snare, and the mass was excised using an argon knife and cryotherapy was administered. Based on microscopic morphology, immune markers, and pathological examination results, the patient was diagnosed with adenoid cystic carcinoma. Bronchoscopy results were reexamined 3 days after the operation; the lumen was unobstructed, and the left main bronchus was clear after resection of the visible neoplasm, and the mucosa was intact within the visible range (**Figure 1B**). After discharge, he was advised to return for a review in 3–6 months; however, he did not follow the doctor’s recommendations. He was admitted to the hospital again due to recurrent cough, expectoration, and wheezing for 1 month, approximately 2 years later, on 5 January 2019. Results of chest CT performed after admission showed occupation of the tracheal and left main bronchus spaces, similar to the previous results (compared with January 2017) (**Figure 2B**). A reexamination of bronchoscopy results on 8 January 2019 revealed obstruction of the left main bronchus due to tumor recurrence; the bronchoscope could not pass through, and the blood supply on the surface of the new organism was obvious (**Figure 1C**). Due to tumor recurrence after 2 years of interventional bronchoscopy, the patient underwent airway neovascularization and ^125^I seed airway stent implantation on 17 January 2019.

Preoperatively, a multiplanar 3D reconstruction was performed based on a thin-layer chest CT. The extent of airway involvement was measured, and the number and distribution of ^125^I seeds (Beijing HTA Co., Ltd., 4.5 mm in length, 0.8 mm in diameter, and 0.50 mCi particle activity) were planned using a treatment planning system (TPS) (Beijing Pheton Holdings Ltd.) (6 particles for the main trachea, 12 particles for the left main trachea, and 6 particles for the right main bronchus), with a dose of 120 Gy prescribed by the planned target volume (PTV). Based on the characteristics of the patient’s tracheal lesions, a Y-shaped covered stent (Sigma-Aldrich; main trachea 18 mm in diameter and 40 mm in length; left main bronchus 12 mm in diameter and 20 mm in length; right main bronchus 14 mm in diameter and 15 mm in length) was selected. Moreover, the Y-shaped covered stent boasts a unique feature that serves as a vessel for ^125^I seeds. The covering acts as a protective layer around the stent, designed to safely house the radioactive seeds. Utilizing specialized forceps, the seeds are meticulously positioned within the covering, ensuring their controlled and secure delivery to the target lesion via the airway stent introducer. The procedure was performed as follows: a rigid scope was inserted under general anesthesia, the pusher was placed along the guidewire under direct endoscopic visualization, and a Y-shaped laminating band stent loaded with 24 radioactive ^125^I seeds was released at the augmentation site, with the position was fixed (**Figure 1D**). For radiation protection, all medical staff wore lead gowns and lead aprons during the procedure. The stent was prepared in a lead box with a leaded glass window and then placed in a metal radiation-resistant stent conveyor, during which the endoscopist wore leaded gloves. Safety-related management of the I-particle Y-laminated stent was conducted according to the standards recommended by the International Commission on Radiological Protection (2). The patient wore lead aprons as required after the procedure, lived in a separate room, and the physicians and nurses who attended to the patient were required to wear lead gowns. Accompanying family members were instructed to maintain a distance of at least 1 m from the patients until the stents containing ^125^I seeds were removed.

**Figure 3:**
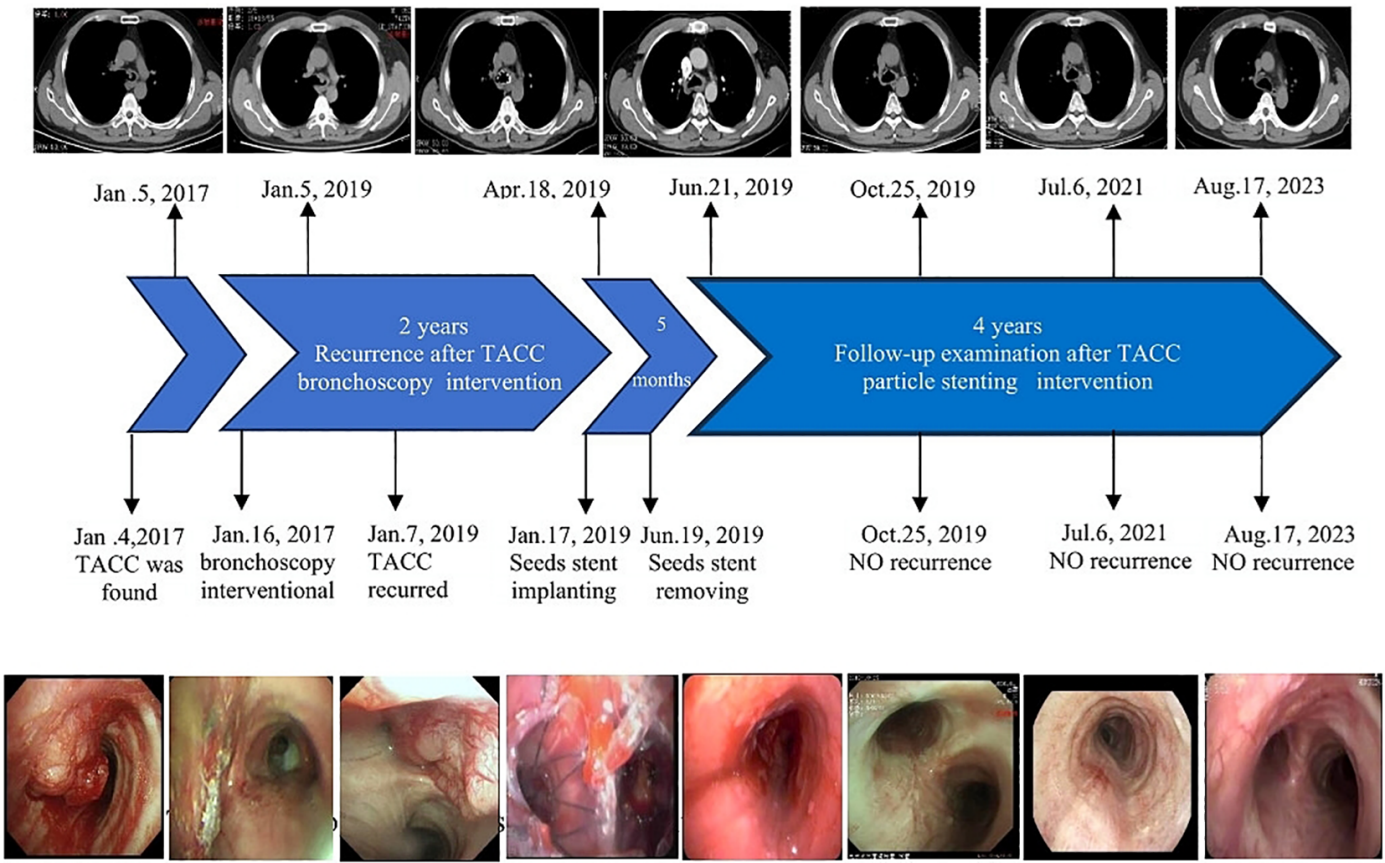
The chronological progression of the entire case.

The patient tolerated the surgery well and had no complications, such as chest pain, persistent cough, hemoptysis, mediastinal emphysema, fistula formation, or secondary pulmonary infection. Bronchoscopy reexamination was performed 3 days later to evaluate the expansion status, position, and whether there were seeds falling off of the stent. Bronchoscopy revealed that the Y-shaped stent was completely attached to the wall, and the seeds remained intact without any displacement. Mucosa congestion and edema were evident within the visible range, and viscous secretions were visible. Sputum was aspirated, revealing a long strip of white necrotic tissue in the lower part of the stent, and was sent for examination. On 18 April 2019, the patient underwent a follow-up chest CT scan, which showed an unobstructed lumen and slight thickening of the left main bronchus wall (**Figures 2C, D**). A reexamination of bronchoscopy results showed that the position of the left stent was fixed, without movement and deformation. There were increased viscous secretions and sputum scabs in the stent, with the mucosa intact in the remaining visible range. Five months postoperatively, on 19 June 2019, ^125^I seed stent removal was performed under general anesthesia. The procedure was as follows: Under general anesthesia, an electronic bronchoscope was inserted through a laryngeal mask. The Y-shaped stents in the left and right main bronchi adhered well. The position was stable with no displacement, although granulation tissue was visible in the lower part of the left main bronchus stent (**Figure 1E**). The laryngeal mask was removed, and a STORZ rigid bronchoscope was inserted using extraction forceps placed through it. The seed stent was completely removed, with all 24 seeds intact and without any displacement. The seed stent was placed in a radiation-proof lead box. A postoperative follow-up chest CT scan on 21 June 2019 revealed thickening of the left wall of the lower trachea whereas the bronchial lumen remained unobstructed (**Figure 2E**). During the follow-up review in October 2019, July 2021, and August 2023, the patient showed no clinical symptoms such as cough and wheezing. On 17 August 2023, a CT scan showed reduced thickening of the left wall of the lower trachea, with no obvious thickening of the left main bronchial wall (**Figure 2F**). Bronchoscopy results revealed that the mucous membrane of the lower tracheal segment and the lateral wall of the left main bronchus was white before the seed stent, with no new lesions, and the mucous membrane was intact in the visible range (**Figure 1F**). The patient was followed up by telephone for 5 years post-seed stent implantation and intracavitary radiotherapy. The patient had no tumor recurrence or clinical symptoms, achieving an 8-year survival. The chronological progression of the entire case is shown in **Figure 3**.

## 3 Discussion

TACC is a salivary gland tumor originating in the submucosal glands of the respiratory tract. Tumors grow in the lumen or along the tracheal wall, with less frequent invasion into the lung parenchyma. It often presents without obvious symptoms in the early stage or with non-specific symptoms such as cough and sputum production. Consequently, the disease is frequently misdiagnosed or missed, with diagnosis often occurring at an advanced stage. Surgical intervention is the treatment of choice for TACC, with primary techniques including tracheobronchial resection with end-to-end anastomosis, carinal and partial lobectomy, carinal resection with reconstruction, lobectomy, laryngotracheal resection with reconstruction, and artificial tracheal placement. However, most TACCs are diagnosed late, with the tumor growing infiltratively along the tracheal wall. The affected area may be more extensive than what is visible to the naked eye. Additionally, due to the length of tracheal resection and the lack of suitable alternative materials, complete resection of the tumor is challenging. For patients who are ineligible for surgery, have positive surgical margins, or have residual tumors after interventional bronchoscopy, radiotherapy can extend survival. However, external radiotherapy can have numerous complications, including radiation esophagitis, pneumonitis, myelitis, tracheal stenosis, tracheoesophageal fistula, and long-term toxicity.

Clinical symptoms at the first visit were non-specific cough and sputum production. The proximity of the tumor to the lower airway and its possible infiltration beyond visible margins made surgical treatment unfeasible. Bronchoscopic argon plasma coagulation, cryotherapy, and other interventional treatments were performed for the first time, yielding favorable short-term results. However, after 2 years, the patient relapsed, experiencing wheezing symptoms, raising the question of an optimal treatment plan for subsequent visits.

In recent years, stents have been used to treat esophageal cancer, malignant biliary obstruction, and hepatocellular carcinoma-induced portal vein tumor thrombosis, demonstrating effective results (3–6). Several studies (3, 7–9) have demonstrated that ^125^I seed stenting effectively reduces jaundice symptoms, improves stent patency, and prolongs survival in patients with malignant biliary obstruction. This technique is a feasible and safe treatment option. A prospective study (10) demonstrated that in patients with advanced esophageal cancer, treatment with ^125^I seed-loaded stents offers slightly longer relief from dysphagia and improved survival compared with conventional covered stents. Xiao et al. (11) evaluated 34 patients with malignant SVCS who underwent endovascular stent implantation in conjunction with ^125^I particle brachytherapy, reporting a median survival of 43.6 weeks (range: 17.1–137.1 weeks). Sun et al. (12) conducted a retrospective analysis of the efficacy of radiotherapy, chemotherapy, and combination therapy for SVCS and found that the median survival was significantly longer in the combination therapy group (stent implantation with radiotherapy and/or chemotherapy) at 36 months, compared with the other three groups.

The stent provides structural support at the stenosis site, whereas ^125^I adsorbed on the stent delivers close-range irradiation to the surrounding tumor tissue to eliminate the tumor. This technique has also been applied to the treatment of airway tumors. Radioactive ^125^I seed airway stents integrate brachytherapy with mechanical airway dilatation by placing radioactive seeds on the surface of ordinary airway stents. The feasibility and safety of inserting radioactive ^125^I seed airway stents have been confirmed in a healthy beagle dog model (13). The first prospective study by Wang (14) showed that ^125^I seed airway stents are safe and feasible for patients with inoperable malignant airway tumor obstruction, significantly reducing airway restenosis and improving overall survival compared with traditional stents.

Although there are limited studies on ^125^I seed tracheal stents for airway tumors, the above studies have confirmed their effectiveness. The patient with TACC experienced tumor recurrence involving the trachea, carina, and main bronchus, leading to the selection of a Y-shaped stent covered with ^125^I. In January 2019, the patient underwent intratracheal tumor resection and ^125^I seed stent implantation using bronchoscopy under general anesthesia. The treatment outcome was favorable. The seed stent was removed 5 months after surgery, and no tumor recurrence was found on chest CT or bronchoscopy 4 years postoperatively. In 2024, the patient was followed up by telephone and reported no respiratory symptoms, with a current survival duration of 8 years. A recent case report (15) demonstrated the successful use of a ^125^I seed airway stent for the treatment of central airway adenoid cystic carcinoma. In this report, the patient underwent implantation of a Y-shaped stainless-steel ^125^I seed in July 2018, which was removed 3 months later. The patient remained recurrence-free for 3 years.

^125^I seed implantation is a form of internal radiotherapy in which radioactive seeds are implanted into the tumor through direct bronchoscopy or CT-guided percutaneous puncture. The treatment principle involves using the treatment planning system (TPS), under the guidance of CT or bronchoscopy, to accurately implant ^125^I radioactive seeds into the tumor target area through the seed implantation puncture needle. Although the radiation energy from radioactive implantation in tumor tissues is relatively low, it can persistently affect and kill tumor stem cells. After receiving a sufficient dose and a corresponding half-life, tumor cells can lose their full proliferative ability, leading to a complete therapeutic effect.

The short penetration distance of the radiation emitted by the seeds prevents damage to the surrounding normal tissues and systemic symptoms. ^125^I seed implantation has been widely used for various cancers, including head and neck cancer, lung cancer, pancreatic cancer, rectal cancer, spinal tumors, and prostate cancer (16). Numerous studies (17–19) indicate that ^125^I seed implantation is more effective for the salvage treatment of recurrent tumors, as it is difficult for patients with recurrence after surgery or radiotherapy and chemotherapy to undergo surgery or external radiotherapy again. The ^125^I stent represents a simple and effective integration of a stent and ^125^I radioactivity, serving the dual purpose of remodeling the lumen and tumor radiotherapy. Furthermore, the short-term placement of ^125^I seed-covered stents can significantly prolong the time to airway restenosis, avoid the need for repeated airway interventional therapy, shorten the treatment cycle, and reduce serious complications. Radioactive ^125^I seed stents offer several advantages for the treatment of malignant airway stenosis (4, 6): rapid relief of dyspnea symptoms, improved quality of life, prolonged survival, effective tumor growth control, reduced occurrence of airway restenosis, high targeting precision, minimal damage to surrounding normal tissues, easy removability and replaceability, with strong flexibility and individual advantages, shorter operation time with less trauma and pain for the patient, and low radiation energy, making them easier to protect and operate on.

This report describes the effectiveness of ^125^I seed stent implantation in the treatment of TACC, confirming its potential as a feasible and innovative option for patients with recurrent TACC. However, this treatment has some limitations. The design and technology of radioactive ^125^I seed airway stents require further improvement. Additionally, the number of particles selected for radioactive ^125^I seed stenting, their activity, and their arrangement in the stent must be standardized. Furthermore, the optimal timing for removing radioactive ^125^I seed airway stents requires further investigation. The clinical efficacy of radioactive ^125^I seed airway stents must be verified in future large-sample studies. In addition, various complications associated with radioactive ^125^I seed stenting should be monitored. The most significant complications are bleeding and airway perforation caused by improper endoscopy. Complications of stenting include chest pain and pneumothorax. Potential complications associated with this treatment method include mediastinal emphysema, subcutaneous emphysema, hemorrhage, irritating choking, stent displacement, stent flattening and fracture, airway restenosis, airway damage, and tracheoesophageal fistulas. Additionally, there is a risk of pulmonary infection and death due to the stent placement.

## 4 Conclusions

For patients with rare tracheal adenoid cystic carcinoma in this case, interventional therapy such as bronchoscopy-assisted argon plasma coagulation and cryotherapy can achieve good results in the short term, but the long-term prognosis needs improvement. ^125^I seed stent implantation may become an important treatment for TACC, but it needs to be further confirmed by multicenter, prospective, large sample randomized controlled study.

## Data Availability

The datasets presented in this study can be found in online repositories. The names of the repository/repositories and accession number(s) can be found in the article/supplementary material.
